# Correction: MHCII reduction is insufficient to protect mice from alpha-synuclein-induced degeneration and the Parkinson’s HLA locus exhibits epigenetic regulation

**DOI:** 10.1038/s41598-025-31393-4

**Published:** 2025-12-29

**Authors:** Elizabeth M. Kline, Janna E. Jernigan, Christopher D. Scharer, Jeffrey Maurer, Sakeenah L. Hicks, Mary K. Herrick, Rebecca L. Wallings, Sean D. Kelly, Jianjun Chang, Kelly B. Menees, Fredric P. Manfredsson, Nikolaus R. McFarland, Jeremy M. Boss, Malú Gámez Tansey, Valerie Joers

**Affiliations:** 1 Department of Physiology, Emory University School of Medicine, Atlanta, GA USA; 2 Department of Molecular and Cell Biology, University of Connecticut, Storrs, CT USA; 3 Department of Neuroscience, University of Florida College of Medicine, Gainesville, FL USA; 4 Center for Translational Research in Neurodegenerative Disease, University of Florida College of Medicine, Gainesville, FL USA; 5 McKnight Brain Institute, University of Florida Health, Gainesville, FL USA; 6 Department of Microbiology and Immunology, Emory University School of Medicine, Atlanta, GA USA; 7 Stark Neuroscience Research Institute, Indiana University School of Medicine, Indianapolis, IN USA; 8 Department of Neurology, Indiana University School of Medicine, Indianapolis, IN USA; 9 Department of Cell Biology, Emory University School of Medicine, Atlanta, GA USA; 10 Parkinson’s Disease Research Unit, Department of Translational Neuroscience, Barrow Neurological Institute, Phoenix, AZ USA; 11 Department of Neurology, University of Florida College of Medicine, Gainesville, FL USA; 12 Norman Fixel Institute for Neurological Diseases, University of Florida Health, Gainesville, FL USA

Correction to: *Scientific Reports* 10.1038/s41598-025-95679-3, published online 21 April 2025

Fredric P. Manfredsson was omitted from the author list in the original version of this Article.

Consequently, in the Author contributions section,

“E.M.K. and M.G.T. conceived the study. E.M.K. designed the study and supervised the technical staff involved in the study. E.M.K., J.J., C.D.S., J.M., S.L.H., M.K.H., R.L.W., S.D.K., J.C., K.B.M., V.J. performed the various surgeries and assays. N.R.M. provided human samples in accordance with IRB. J.M.B. and M.G.T. obtained funding support. J.M.B. and C.D.S. provided guidance and interpretation on ATAC-seq and MHCII gene expression. E.M.K., J.J., M.G.T. and V.J. wrote and edited the paper. E.M.K., J.J. and V.J. were responsible for all prepared figures. All authors reviewed and approved the final version of the manuscript.”

now reads:

“E.M.K. and M.G.T. conceived the study. E.M.K. designed the study and supervised the technical staff involved in the study. E.M.K., J.J., C.D.S., J.M., S.L.H., M.K.H., R.L.W., S.D.K., J.C., K.B.M., F.P.M., and V.J. performed the various surgeries and assays. N.R.M. provided human samples in accordance with IRB. J.M.B. and M.G.T. obtained funding support. J.M.B. and C.D.S. provided guidance and interpretation on ATAC-seq and MHCII gene expression. E.M.K., J.J., M.G.T. and V.J. wrote and edited the paper. E.M.K., J.J. and V.J. were responsible for all prepared figures. All authors reviewed and approved the final version of the manuscript.”

Furthermore, the Acknowledgements section has been updated. It now reads:

“We thank the Emory Flow Cytometry Core for their assistance with the use of the LSRII, as well as the Emory Genomics Core for completing library preparations and sequencing. We thank Haydeh Payami and the Tansey lab for thoughtful discussions. Partial funding from this work came from a pilot grant from Emory PD-CERC (MGT and JMB), a pilot grant from NIH/NINDS 1P50NS071669 Emory’s Udall Parkinson’s Disease Center/UL1RR025008 Atlanta Clinical and Translational Science Institute (ACTSI) (JMB and MGT), NIH RO1AI153102 (JMB), NIH/NINDS RF1NS128800 (MGT) and NIH/NINDS 5R01NS092122 (MGT and JMB). Support was also provided by Michael J Fox foundation MJFF-023296 (VJ and MGT), 1FL ADRC Alzstars P30AG066506 (VJ) and Alzheimer’s Association AARG-22-927012 (VJ). Figure 1 was created in BioRender. Jernigan, J. (2025) https://BioRender.com/r71q005.”

In addition, the affiliation “Department of Physiology, Emory University School of Medicine, Atlanta, GA, USA” was omitted for authors Elizabeth M. Kline, Mary K. Herrick, Sean D. Kelly, Jianjun Chang, Malú Gámez Tansey, and Valerie Joers.

The original Article has been corrected.

