# Supplementary material for: MHCII reduction is insufficient to protect mice from alpha-synuclein-induced degeneration and the Parkinson’s HLA locus exhibits epigenetic regulation
Source: Sci Rep. 2025 Apr 21;15:13705. doi: 10.1038/s41598-025-95679-3 (PMC12012047; doi:10.1038/s41598-025-95679-3)

# Uncropped original western blots

TH western from figure 5

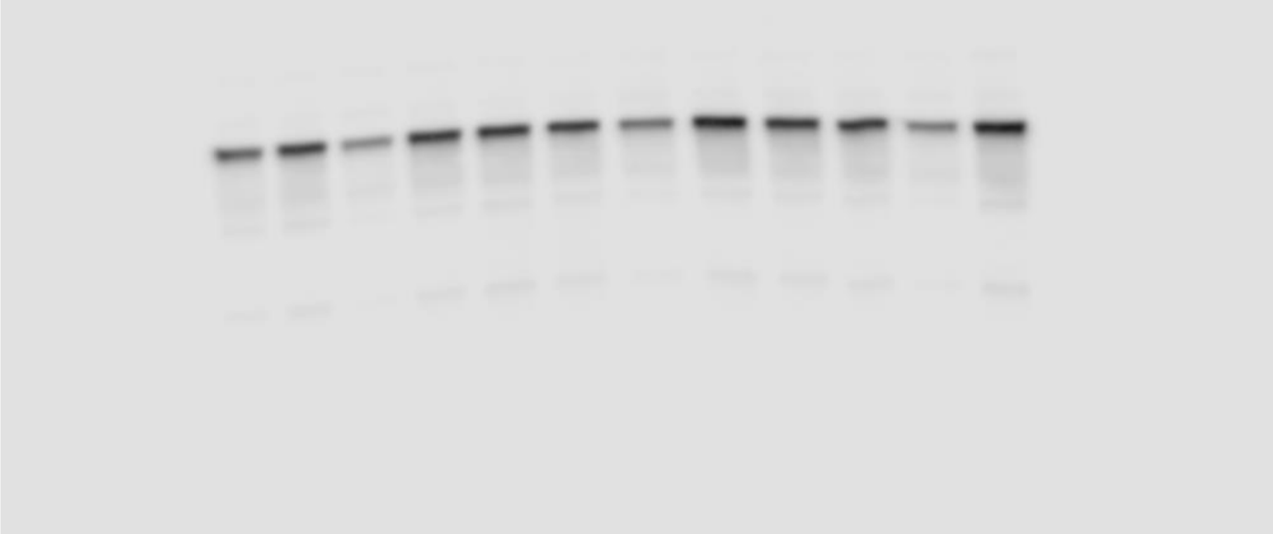

Total protein stain for TH blots from figure 5

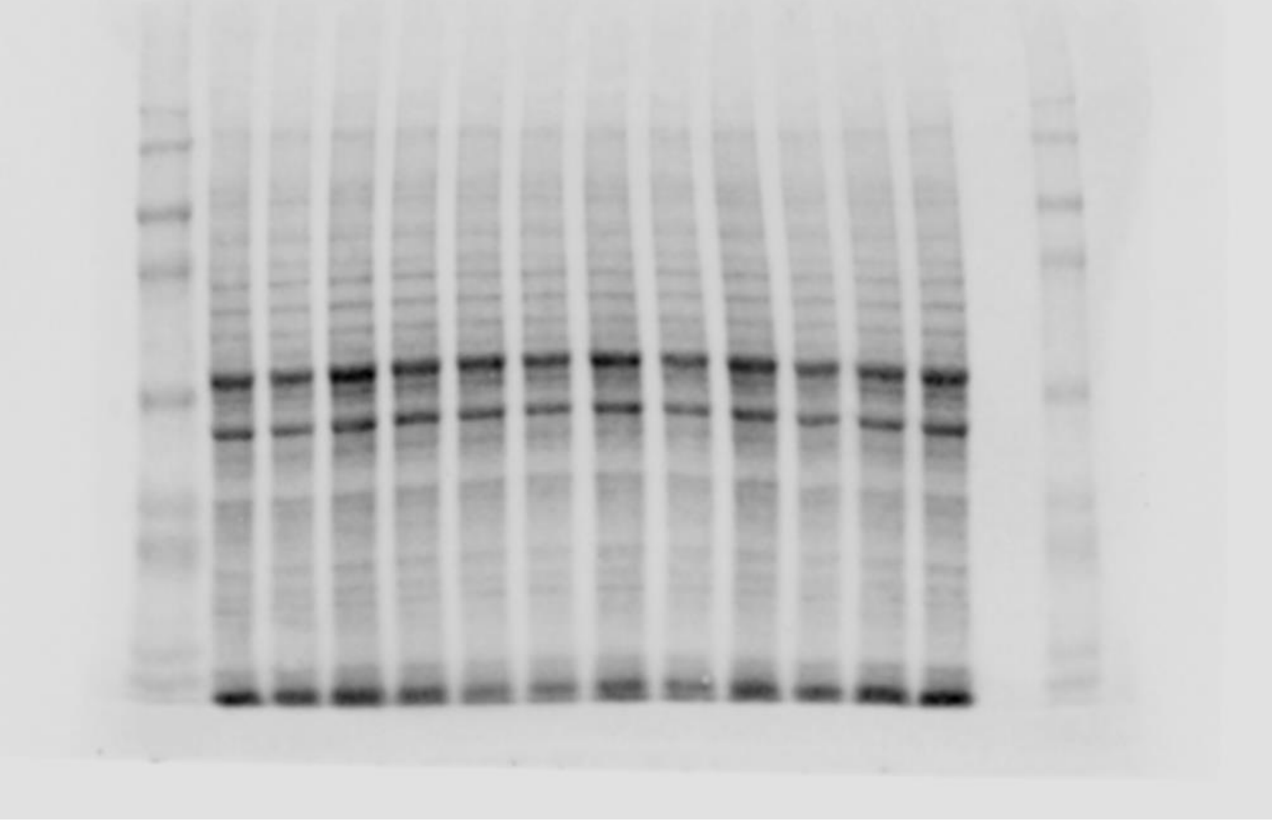

DAT western from figure 5

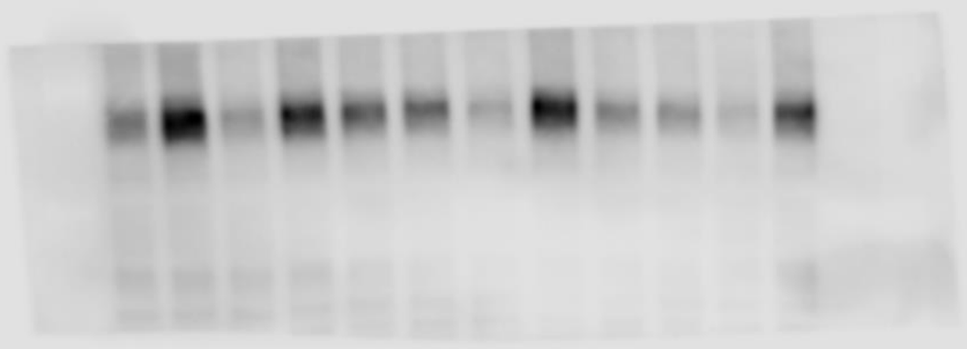

pTH western from figure 5

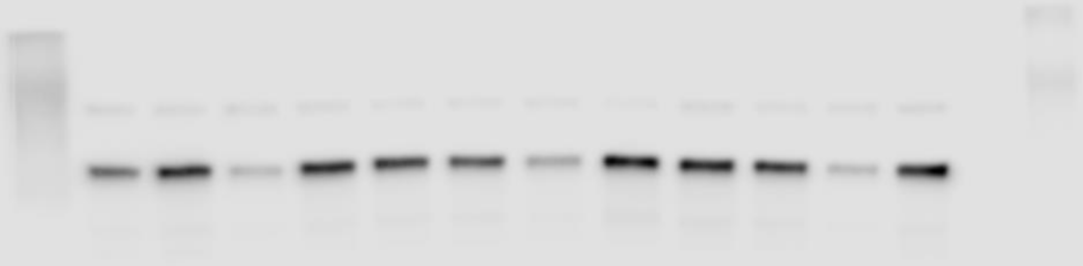

Total protein stain for DAT
and pTH blots from figure 5

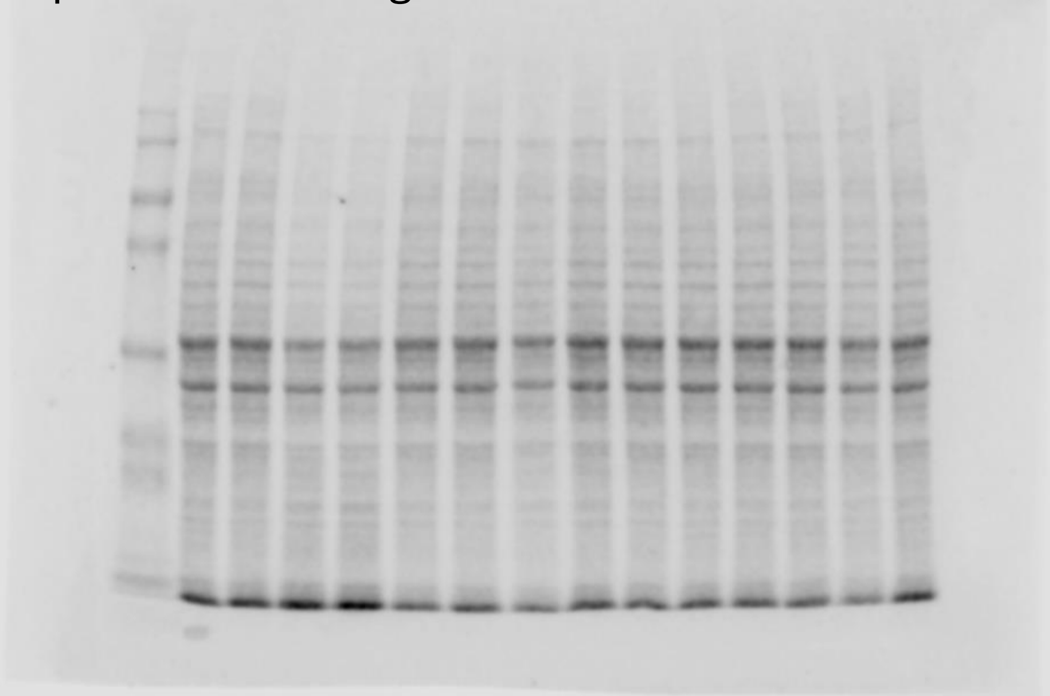

Human alpha-synuclein (Syn211)
western from supplemental figure 2

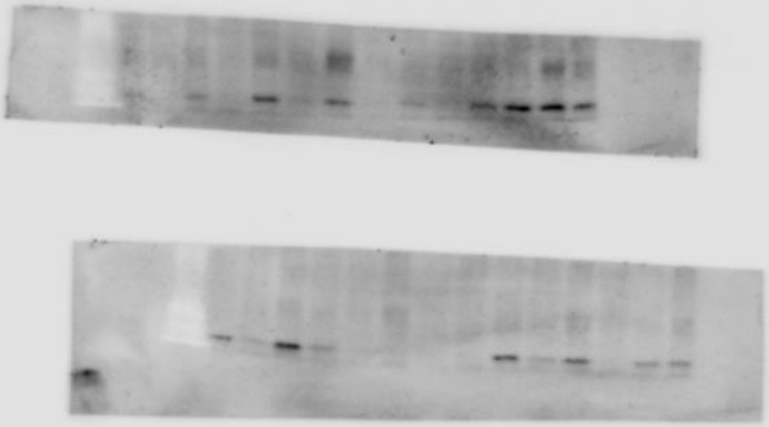

Supplement: Supplementary file 2 — Supplementary Material 2 [file 41598_2025_95679_MOESM2_ESM.pdf]
